# How Do Outpatients Experience 20‐Session Cognitive‐Behavioral Therapy for Anorexia Nervosa (CBT‐AN‐20)? A Qualitative Exploration

**DOI:** 10.1002/eat.24528

**Published:** 2025-08-21

**Authors:** Heather C. Duggan, Charlotte Rose, Hannah Turner, Jessica Cox, Rachel Ebbens, Jade Southron, Glenn Waller

**Affiliations:** ^1^ University of Sheffield, School of Psychology Sheffield UK; ^2^ Avon and Wiltshire Mental Health Partnership NHS Trust Bristol UK; ^3^ Hampshire and Isle of Wight Health NHS Foundation Trust Southampton UK

**Keywords:** anorexia nervosa, CBT, cognitive‐behavior therapy, eating disorders, evaluation, outpatient, patient experience, qualitative

## Abstract

**Objective:**

More efficient psychological treatments are needed to move patients with anorexia nervosa toward recovery at the earliest opportunity and reduce treatment waiting times. CBT‐AN‐20 is a novel, 20‐session cognitive‐behavioral therapy for outpatients with anorexia nervosa, emphasizing recovery from the very start of treatment. This qualitative study explored adult patients' experiences of receiving CBT‐AN‐20.

**Method:**

Sixteen patients' survey data were collected (January 2023–May 2024) as part of a broader study conducted in two UK‐based specialized community eating disorder services (Preregistration: https://doi.org/10.17605/OSF.IO/6RTSK). Patients were adults with anorexia nervosa assessed as suitable for outpatient therapy. Respondents included treatment completers and non‐completers. Responses were analyzed using descriptives (Likert‐scale questions) and thematic analysis (open‐ended questions). Ethical clearance was obtained.

**Results:**

CBT‐AN‐20's suitability was rated moderately high, and its helpfulness was rated positively. Thematic analysis identified three main themes: “Importance of Therapeutic Relationship”, “Characteristics of Therapy” (i.e., its structure and content), and “Experiences of Therapy Over Time”. While patients broadly perceived the therapeutic relationship as important, they had mixed opinions regarding its characteristics, and their experiences of therapy changed over time and at different treatment stages.

**Discussion:**

CBT‐AN‐20 appears acceptable and potentially helpful for some adult outpatients with anorexia nervosa. The development of targeted psychoeducational materials to motivate patients through CBT‐AN‐2's challenging early stages is recommended. Future research should involve patients in the qualitative analysis and explore therapists' perspectives. Triangulating these results with patient outcome data will enable researchers to evaluate the next steps in developing and testing this novel therapy.

**Summary**

- This qualitative study explored adult patients' experiences of receiving CBT‐AN‐20, a novel, 20‐session cognitive‐behavioral therapy for outpatients with anorexia nervosa.
- Thematic analysis identified three main themes: “Importance of Therapeutic Relationship”, “Characteristics of Therapy”, and “Experiences of Therapy Over Time.”
- CBT‐AN‐20 appears to be an acceptable and potentially helpful treatment for some adult outpatients with anorexia nervosa.
- Triangulating these results with patient outcome data will enable researchers to evaluate the next steps in developing and testing this novel therapy.

## 1 Introduction

More efficient psychological treatments are needed for patients with anorexia nervosa. Among other options, the UK National Institute for Health and Care Excellence (NICE 2017) recommends up to 40 sessions of cognitive‐behavioral therapy (CBT) (Fairburn 2008), contributing to long waiting times (Beat 2021). To address this problem, a novel, 20‐session CBT has been developed for outpatients with anorexia nervosa (CBT‐AN‐20), with greater emphasis on early change and exposure therapy (among other CBT skills). Preliminary outcomes (Duggan et al. 2025, under consideration) are comparable to those of 40‐session CBT‐E.

Exploring patients' experiences of CBT‐AN‐20 is an important part of evaluating its feasibility and acceptability at this early stage of intervention development, allowing for the triangulation of quantitative and qualitative data to ensure that future iterations of CBT‐AN‐20 meet patients' needs (de la Rie et al. 2006). Understanding patients' experiences can explain patient engagement, retention, and benefit from the therapy. Thus, this approach can highlight aspects of this treatment that require further research and development in order to further support more patients in recovering from their eating disorder.

Previous evaluations of psychotherapies for eating disorders have used thematic analysis to understand the experiences of treatment for patients with anorexia nervosa (Isaksson et al. 2021; Lose et al. 2014; Rennick et al. 2024; Zainal et al. 2016) and non‐underweight eating disorders (Hoskins et al. 2019; McClay et al. 2013; Sánchez‐Ortiz et al. 2011; Toro et al. 2023; Traviss et al. 2011). Common themes include the therapeutic relationship, the characteristics of therapy (i.e., its structure and content), and treatment outcomes (Supporting Information A). However, none of those previous studies investigated the experiences of patients with anorexia nervosa in CBT.

The current study aimed to explore patients' experiences of CBT‐AN‐20 during its early stages of intervention development and testing, using survey data collected from two National Health Service (NHS) outpatient eating disorder services in the United Kingdom. It was part of a wider study to evaluate CBT‐AN‐2's feasibility, acceptability, and preliminary effectiveness (Duggan et al. 2025, under consideration). Empirical results from these two NHS services indicated high feasibility (60 patients recruited), moderate acceptability (22 completed treatment), and good patient outcomes, with significant improvements in eating disorder symptoms and weight, and large effect sizes comparable with Fairburn et al. (2013) and Jenkins et al. (2019).

The hypothesis‐generating research questions guiding the current study data analysis were:

1. What are patients' experiences of CBT‐AN‐20?
2. What do patients' experiences tell us about how we might improve CBT‐AN‐20?

## 2 Method

This study was preregistered (https://doi.org/10.17605/OSF.IO/6RTSK) and O'Brien et al.'s (2014) Standards for Reporting Qualitative Research (SRQR) were followed in writing this paper.

### 2.1 Design

This study has a survey design. A survey was chosen as a pragmatic method of collecting qualitative data from as many patients as possible in busy outpatient services as a first step in evaluating patients' experiences of CBT‐AN‐20, in line with previous research (Hoskins et al. 2019; Toro et al. 2023; Zainal et al. 2016). To capture a full range of experiences, all 41 patients who received CBT‐AN‐20 were sent an email inviting them to take part in the research via an online survey, regardless of completion or early ending. The invitation came from the relevant head of clinical service (authors H.T. or C.R.), rather than the therapist who had seen the patient, to reduce any risk of demand characteristics (e.g., the patient feeling that they could not be honest with their therapist). There were no set times between the end of therapy (completion or early ending) and the invitation to take part in the study, as the data were collected over a limited time period, rather than as the clinical work progressed. The number of patients who returned completed surveys dictated the sample size. A pragmatic approach was also taken to the data analysis, meaning that the methods chosen were those deemed most appropriate to address the research questions (Allemang et al. 2022; Feilzer 2010). Therefore, descriptive statistics were chosen to analyze quantitative responses (Likert scales), while thematic analysis was chosen to identify themes across patients' qualitative responses.

### 2.2 Ethics

This study was cleared under UK NHS Research and Development regulations, whereby monitoring good practice and service improvement does not require ethical clearance. Patients were provided with an information sheet about the purpose of the study and consented by ticking the relevant box once they logged on to the Qualtrics survey. They were informed that they could discuss questions about the study with their therapist. Patients consented to data collection and to the sharing of anonymized data and verbatim quotes with the Sheffield research team (H.C.D. and G.W.) for analysis and inclusion in research publications. Ethical permission for data analysis (including the use of anonymized quotes from the participants) was obtained from the University of Sheffield (064046).

### 2.3 Intervention: CBT‐AN‐20

CBT‐AN‐20 is a 20‐session therapy with similar structure, content, and delivery to CBT‐T (Waller et al. 2019), adapted for treating anorexia nervosa (Waller et al. 2025). It is a formulation‐based approach, which targets: early weight restoration and eating normalization based on nutrition and exposure (Phase 1); weight regain/stabilization (Phase 2); emotional triggers and maintenance (Phase 3); body image (Phase 4); and relapse prevention (Phase 5). Unlike other CBT‐ED (e.g., Fairburn 2008), CBT‐AN‐20 contains a lot of exposure. Specifically, it follows the inhibitory learning approach (Craske et al. 2014), which encourages patients to experience their maximally tolerable levels of anxiety because this supports faster learning when facing their fears (e.g., eating more, regaining weight). Given the importance of early change (Vall and Wade 2015), early use of motivational work and addressing the'anorexic voic' are included as needed, and progress is reviewed at session 6. If patients are making limited progress (particularly early weight regain), they are invited to return to treatment when able to engage in change or are offered alternative/more intensive treatment.

Upon presentation to one of the two participating NHS services, patients were diagnosed using full DSM‐5 criteria for anorexia nervosa, based on a diagnostic assessment (clinical interview) by a qualified clinician. Only patients with a BMI over 15 were included (as in Fairburn et al. 2013), to ensure safety to undertake psychological therapy. Diagnoses were reviewed for accuracy at the start of treatment, and all patients met the full criteria for anorexia nervosa. CBT‐AN‐20 therapists were predominantly Assistant Psychologists (trained and supervised by experienced psychological therapists) in two NHS outpatient services.

### 2.4 Participants

Eligible patients were adults diagnosed with anorexia nervosa, based on BMI = 15–19 plus clear starvation features (Calugi et al. 2017; Keys et al. 1950), including physical, emotional, cognitive, and behavioral symptoms (e.g., cold intolerance, fatigue, low mood, food preoccupation, social withdrawal). They were a case series of consecutive patients referred to the two NHS services who were identified as eligible to receive CBT‐AN‐20 and consented to receive this treatment as part of routine outpatient services (Duggan et al. 2025, under consideration). No control groups were included. All were assessed as suitable for outpatient eating‐disorder‐focused therapy (i.e., not requiring hospitalization, actively suicidal, or engaging in serious self‐harm). Patients with diagnosed or suspected autistic spectrum disorder were excluded from this study, though they will be included in future iterations when it is clearer that existing therapies such as CBT are suitable or can be adapted for this group (e.g., Tchanturia et al. 2020). Eating disorder duration was not an exclusion criterion, given the evidence that those with longer duration of anorexia nervosa are able to benefit equally from evidence‐based treatment (e.g., Raykos et al. 2018; Radunz et al. 2020). Participation in this survey was not mandatory for receiving therapy. Sixteen (39.03%) of the 41 patients took part.

### 2.5 Procedure

An online survey (Supporting Information B) was created on Survey Monkey/Microsoft Forms to collect patients' demographics, clinical characteristics, and responses to quantitative and qualitative questions. Surveys were designed with input from people with lived experiences of eating disorders to ensure questions made sense from a patient perspective and were emailed to patients by two of the authors (C.R. and H.T.), rather than by their therapists, to facilitate open and honest feedback. Patients rated their experience of CBT‐AN‐20 on eight questions (e.g., “How much do you feel the treatment helped you to reduce your eating disorder behaviors?”), rated 0 (not at all) to 10 (completely), followed by seven open‐ended questions collecting narrative responses (e.g., “What did you find most helpful about treatment and why?”).

### 2.6 Data Analysis

Descriptive statistics (Microsoft Excel v.16.99.1) summarized patients' demographics, clinical characteristics, and responses to Likert‐scale questions.

Thematic analysis was used for qualitative responses, based on Braun and Clarke's (2006) six‐step process (data familiarization, generating initial codes, searching for themes, reviewing themes, defining/naming themes, and producing the report). The main analyst (H.C.D.) coded a Word document containing participants' open‐ended qualitative responses to the survey questions using a predominantly inductive, semantic approach to capture the explicit meaning of patients' responses. H.C.D. completed two rounds of coding, creating codes from the data rather than using a predesigned coding list, before creating an Excel table of the codes and quotes. An iterative approach was used to refine the codes, identify and review potential themes, and generate a candidate thematic map. Codes were printed on paper, and H.C.D. grouped them into potential themes, which were then mapped out in Word and Excel for quantification and further development. As part of this process, codes were also further revised by making amendments, adjustments, and aggregations. At the fifth iteration, a candidate map was developed.

A second analyst (J.S.) with lived experience independently assessed whether the candidate themes accurately represented patients' responses. This was achieved by comparing the Word document with participants' qualitative data with the proposed themes and using a quote‐matching activity (Supporting Information C and D). Percentage agreement (Zhao et al. 2022) and Cohen's Kappa (Cohen 1960; McHugh 2012) delivered interrater reliability (IRR; Supporting Information C). There was 87.93% IRR of matching quotes to themes, 65.52% to subthemes, and 82.76% to sub‐subthemes (Cohen's Kappa = 0.44, 0.25, and 0.41, respectively) indicating fair‐to‐moderate agreement (Cohen 1960). Discrepancies were discussed to achieve 100% agreement before H.C.D. revised the thematic map. J.S. and H.C.D. agreed upon the eighth iteration of the thematic map as the final version of the analysis.

#### 2.6.1 Reflexivity

H.C.D. and J.S. have some experience in thematic analysis. H.C.D. is a PhD researcher with academic knowledge of CBT‐AN‐20 as her research topic. J.S. works with eating disorder patients but had no prior experience with CBT‐AN‐20. Their data analysis adds a fresh perspective, as neither analyst was involved in CBT‐AN‐2's design, delivery, or data collection. Both analysts are White females with lived experience of an eating disorder. This insider positionality offers a deeper understanding of patients' experiences and the nuances of their conflicting emotions (Berger 2015; Dwyer and Buckle 2009). It also inherently shapes the analysts' interpretations of participants' responses, potentially limiting objectivity. Both analysts have existing relationships with the team that developed CBT‐AN‐20, which may introduce a potential source of bias. H.C.D. is supervised by G.W., and J.S. works in the same service as C.R. However, neither analyst was involved in developing CBT‐AN‐20, delivering therapy, or data collection for this study, and J.S.'s independence from the CBT‐AN‐20 project counteracts potential biases introduced by H.C.D.'s insider‐outsider positionality, supporting the results' credibility.

## 3 Results

### 3.1 Participant Characteristics

Sixteen patients (40% response rate) completed surveys between January 2023 and May 2024. Table 1 reports demographics and clinical characteristics. Nine (56.25%) were aged 24 or under, and 15 (93.75%) were employed/in education. The sample included patients who had previously received therapy, meaning some could compare prior experiences with undertaking CBT‐AN‐20. Patients reported attending 4–23 sessions (mean = 14.77, SD = 7.37), reflecting the inclusion of both treatment completers and non‐completers. Data were not available to compare baseline characteristics of survey responders and non‐responders.

**TABLE 1:** Participant demographics.

Characteristic	*n* (%)
Age range (years)	
18–24	9 (56.25%)
≥ 25	7 (43.75%)
Ethnicity	
White	13 (81.25%)
Sex	
Female	15 (93.75%)
Gender identity	
Women	15 (93.75%)
Previous therapy for eating disorder	
Yes	6 (37.50%)
No	10 (62.50%)
If yes, number of previous courses of therapy	
1–3	5 (31.25%)
No. sessions of CBT‐20‐AN attended	
< 20	7 (43.75%)
≥ 20^a^	6 (37.50%)
Unknown	3 (18.75%)
Completer status^b^	
Completer	9 (56.25%)
Non‐completer	7 (43.75%)

*Note: N = 16*. Demographics are presented for groups of no fewer than five participants to protect anonymity.

^a^ A full course of CBT‐AN‐20 is 20 sessions plus one assessment and up to two follow‐up sessions (up to 23 sessions in total).

^b^ Completers were defined as patients who had completed a full course of therapy, or fewer in some cases, and were identified by therapists and supervisors. This is why we have nine completers (six who received 20 sessions or more of CBT‐AN‐20, and three who completed in fewer than 20 sessions). Non‐completers were defined as patients who left therapy early, whether through their own choice or being asked to leave therapy early due to lack of active engagement.

### 3.2 Quantitative Analysis

Table 2 presents the means and ranges of patients' responses to the eight Likert‐scale questions (rated 0–10) (see also Supporting Information E). CBT‐AN‐20 suitability was rated moderately high, and helpfulness across a range of outcomes was rated positively. The treatment was rated lowest for improvements in body image and highest for helping patients move toward recovery. Patients were relatively likely to recommend CBT‐AN‐20 to others with similar eating difficulties.

**TABLE 2:** Patient responses (*N* = 16) to quantitative survey questions.

Quantitative survey responses	*M* (range)
How suitable did the treatment feel for you as an individual?^a^	7.00 (1–10)
How much do you feel the treatment helped you to reduce your eating disorder behaviors?^a^	6.31 (0–10)
How much do you feel the treatment helped you to reduce your eating disorder thinking patterns?^a^	6.00 (0–10)
How much do you feel the treatment helped you to improve your body image?^a^	5.38 (0–10)
How much do you feel the treatment helped you to improve your emotional state?^a^	6.80 (0–10)
How much do you feel the treatment helped you move toward recovery?^a^	6.88 (0–10)
How much do you feel the treatment helped you to improve your quality of life?^a^	6.50 (0–10)
How likely is it that you would recommend this therapy to someone with similar eating difficulties to you?^b^	7.33 (1–10)

^a^ Likert scale: 0 = not at all, 10 = completely.

^b^ Likert scale: 0 = not at all likely, 10 = extremely likely.

### 3.3 Thematic Analysis

**FIGURE 1:**
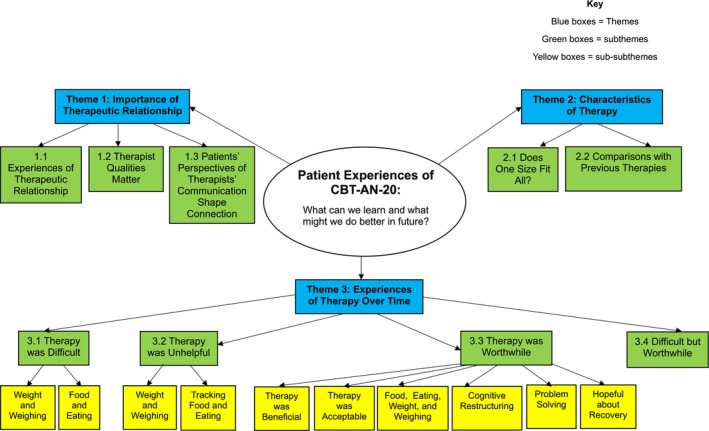
Thematic map.

Figure 1 maps a thematic analysis of patients' qualitative survey responses. Three broad themes were identified: “Importance of Therapeutic Relationship,” “Characteristics of Therapy,” and “Experiences of Therapy Over Time.” Subthemes and sub‐subthemes identify nuances within the broader themes, providing deeper insight into patients' experiences of CBT‐AN‐20. These are illustrated with quotes from each theme in Tables 3, 4, 5. Patients' identification numbers are given alongside examples of quotes, showing that responses were from across the dataset.

**TABLE 3:** Illustrative quotes from each subtheme in the “importance of therapeutic relationship” theme.

Subtheme	Nature of contribution	Illustrative quotes	Patient number
1. Experiences of therapeutic relationship	Positive	“I had a very good relationship with my therapist”	14
		“I had built a good relationship with the staff member, and her overall approach to my eating disorder helped me mentally and emotionally”	6
	Negative	“Terrible. There wasn't one (a relationship)”	1
2. Therapist qualities matter	Knowledge/expertise and non‐judgmental qualities	“The sessions with her felt experienced and expert. She was equipped to initiate problem solving and implement changes”	2
		“My therapist was never judgmental and always provided me with an answer to all my questions”	12
	Interest, empathy and safety	“She was very personal and treated me with respect and as an individual”	6
		“She was incredibly empathetic to me and inspired so much hope in my recovery”	5
		“She made me feel really safe and not to feel ashamed of my condition”	16
	Negative/feared qualities	“I couldn't fully express everything I wanted to out of fear of judgment”	7
		“I had no faith in her, and I think that is why I wanted to leave treatment early”	1
3. Patients' perspectives of therapists' communication shape connection	Importance of feeling heard	“I was comfortable to talk freely with them. I felt heard and understood”	14
		“I got on well with my therapist and felt she listened to my concerns and difficulties”	11
		“It was like talking to a robot”	1
		“I felt slightly disregarded and some of my issues de‐validated”	7
	Importance of feeling comfortable with the therapist	“I never felt afraid to speak openly, knowing that my team [at the NHS service] held no judgment but would rather help me through the problem to continue on to success”	5
		“I was originally pretty hesitant to open up, but as I became more comfortable, it felt easier”	14

**TABLE 4:** Illustrative quotes from each subtheme in the “characteristics of therapy” theme.

Subtheme	Nature of contribution	Illustrative quotes	Patient number
1. Does one size fit all?	Tailored to my needs	“It was helpful to guide my treatment around what we both felt was benefiting me the most”	5
		“She was able to relate to this [managing grief alongside an eating disorder] and take this into consideration during our sessions”	6
	Not tailored to my needs	“To be more tailored to the individual and the non‐negotiable be reassessed as one glove does not fit all”	3
		“Less focus on weight, more focus on the individual, rather than trying to shove them [patients] into a pre‐made framework that is presumed to work for everyone”	1
	Needs more on comorbidity	“I have a lot of other inter‐related issues, and it would have been very helpful to address them alongside instead of having to focus exclusively on the eating disorder”	13
		“I would prefer a more individual‐based approach which is more flexible and trauma‐informed”	8
2. Comparisons with other therapies	Better than other therapies	“This was orders of magnitude better than any other therapy I've had.”	13
		“Miles better than any therapy I've received from any other services”	5
	Worse than previous therapies	“Previous experiences were better. They were informative and scientific (I find it easier to challenge thoughts if there is proper evidence to back it up), and the therapist actually listened and behaved like a human rather than being condescending and patronizing”	1
		“I found my previous forms of therapy more beneficial to me as they tackled the past traumas that then encouraged me to tackle the current behaviors, etc. However, I found this one was not attacking the root—just the surface‐level issues”	7
	Novel approach	“[Previously] I only had a therapist, but it was not specifically focused on food. Therefore, it was different”	16
		“Treatment felt like a newer approach with many aspects I had never encountered before”	5

**TABLE 5:** Illustrative quotes from each subtheme in the “experience of therapy over time” theme.

Subtheme	Nature of contribution/sub‐subtheme	Illustrative quotes	Patient number
1. Therapy was difficult	Weight and weighing	“It was the focus on weight gain each week I found difficult. It allowed the demon in my head to take control”	6
		“Sometimes I struggled to gain weight, and I was really stressed to be on the same weight for around 2 months eating more”	16
	Food and eating	“The more I thought about food in the moment, the more negative thoughts I had, and it triggered more anxieties as I was having to overthink the food”	3
		“Having to write down everything I ate and drank made me want to limit my intake”	14
2. Therapy was unhelpful	Weight and weighing	“I hated the fact that weighing is compulsory ‐ even if you don't think that you will be affected by knowing it [weight], the numbers play on your mind”	1
		“I had to reach weight progression targets to be able to continue with the therapy program. I found the pressure of this made my eating disorder put up barriers. It took control”	6
	Tracking food and eating	“I found food diaries particularly difficult as tracking food was what caused my eating disorder to spiral initially”	11
		“In some ways, I felt abnormal doing this [tracking] as a normal person wouldn't, and I wanted to be as normal as possible.”	3
	Exposure elements	“The exposure to everything [was unhelpful]: knowing my weight consistently, constantly tracking my food intake and being vulnerable to seeing the shape and size of my body in such an uncomfortable setting and format”	7
3. Therapy was worthwhile	Therapy was acceptable	“I would have liked to complete the whole treatment [i.e., additional treatment to work toward full recovery] with [my therapist]”	15
		“It helped more than I thought it would. And I managed a lot more than I thought.”	10
		“If I had gone to the stage [of therapy] of speaking about self‐image, I would've benefited.”	3
	Therapy was beneficial	“I found the therapy to be really beneficial. Although my weight did not reflect this, the sessions overall helped my confidence”	6
		“Overall, I think the course is effective, especially if you get along with your therapist.”	14
		“I have noticed a significant shift in my attitudes toward food and allowing myself to challenge fear foods as well as my thought processes”	11


	“It greatly helped me to regain valuable aspects of my life, including socially and professionally”	9
	Value of work on food, eating and weight	“I found the discussions around how I could improve my food intake very beneficial”	6
		“I found the regular check‐in on weight helpful to know I was on the right track”	7
	Value of cognitive restructuring	“[Therapy] provided me with the knowledge on why I thought and did the things I did. All the resources were very useful, and I actually enjoyed learning about myself through them”	12
	Value of problem solving	“[My therapist] truly understood where everything came from and tried to explain to me how to cope and solve the problem”	16
Hopeful about recovery	“Changing a core belief takes time, and I'm still working on them now, outside of the CBT treatment. But I wouldn't have been able to start this change without the treatment”	14
	“Positive [feelings about therapy] and helped me feel that recovery is possible”	15
4. Difficult but worthwhile	N/A	“Whilst it was very difficult at times, I'm glad I persevered”	9
		“It was very difficult to have to put on weight and give up control of my eating, weight and shape. They were what made me feel safe in the world, and giving them up was extremely traumatic and distressing. It caused me to have suicidal thoughts. It's the worst experience I've had in my life. I couldn't even have imagined anything so awful before. Now I'm out the other end, it was absolutely worth it”	13

#### 3.3.1 Theme 1: Importance of Therapeutic Relationship

This theme includes 12 (75.00%) patients' perspectives of the therapeutic alliance and its importance in shaping their therapy experiences. It consisted of three sub‐themes, as shown in Figure 1—Experiences of therapeutic relationship (11/16 patients—68.75%); Therapist qualities matter (10/16 patients—62.5%); and Patients' perspectives of therapists' communication shape connection (13/16 patients—81.25%). Each is illustrated with sample quotes in Table 3.

#### 3.3.2 Theme 2: Characteristics of Therapy

This theme contains 12 (75.00%) patients' comments on CBT‐AN‐2's structure and content. Patients reported mixed opinions regarding the extent to which they felt individualization of therapy mattered and how CBT‐AN‐20 compares with other psychotherapies. These are captured in the two sub‐themes that emerged—Does one size fit all (7/16 patients—43.75%); and Comparisons with other therapies (6/16 patients—37.5%). Each is illustrated with sample quotes in Table 4.

#### 3.3.3 Theme 3: Experiences of Therapy Over Time

This theme involved 15 (93.80%) patients' changing perspectives of CBT‐AN‐20 over the course of therapy, reflecting its greater complexity over time (see Table 5). Some patients reported finding aspects of CBT‐AN‐20 ‘difficult’ (11/16; 68.75%), with many non‐completers retaining this view of therapy as ‘unhelpful’ (8/16, 50%). In contrast, completers' experiences developed into a dialectical perspective of therapy as “difficult but worthwhile” (6/16; 37.5%). Critically, the majority of both completers and non‐completers identified beneficial aspects of therapy (13; 81.25%), particularly in the focus on food and weight, the use of cognitive restructuring and the focus on problem‐solving, resulting in “hopefulness for recovery.” Hence, the number of patients who found elements of therapy positive and negative often added to more than 100%, reflecting different points in patients' therapeutic journeys.

## 4 Discussion

This study is the first to explore patients' experiences of CBT‐AN‐20, a novel 20‐session intervention for outpatients with anorexia nervosa. Quantitative analyses indicate that CBT‐AN‐20 is experienced as suitable and helpful by some patients and not others; the thematic analysis of patients' qualitative responses elaborated on that pattern. While patients broadly perceived the therapeutic relationship as important, they reported mixed experiences of its characteristics. Furthermore, patients' experiences of therapy changed over time and across treatment stages, potentially explaining why non‐completers broadly found CBT‐AN‐20 difficult and unhelpful (albeit with some helpful aspects), while completers found it difficult but worthwhile.

### 4.1 Comparison With Previous Studies

Results are broadly comparable with previous studies of patients' experiences of psychotherapies for eating disorders (Supporting Information A). Similar themes were identified, including: therapeutic relationships (Hoskins et al. 2019; Isaksson et al. 2021; Lose et al. 2014; McClay et al. 2013; Toro et al. 2023; Traviss et al. 2011); changing experiences of therapy over time (Hoskins et al. 2019); and similar mixed opinions regarding therapy duration (Hoskins et al. 2019; Lose et al. 2014; Rennick et al. 2024; Zainal et al. 2016), individualization (Hoskins et al. 2019; Zainal et al. 2016), and comparisons with previous therapies (Hoskins et al. 2019; Lose et al. 2014; Sánchez‐Ortiz et al. 2011; Zainal et al. 2016). Patients report positive and negative experiences of CBT‐AN‐20, as in studies of other evidence‐based eating disorder psychotherapies (e.g., Hoskins et al. 2019; Lose et al. 2014; Sánchez‐Ortiz et al. 2011; Toro et al. 2023; Zainal et al. 2016), reflecting individual differences in therapy experience.

Notably, the present results most closely resemble Hoskins et al.'s (2019) qualitative evaluation of patients' experiences of CBT‐T—a similar therapy to CBT‐AN‐20 for non‐underweight patients with eating disorders, sharing an emphasis on exposure and early change. Patients in both studies reported similar positive therapist qualities (caring, understanding, approachable, knowledgeable, capable of problem‐solving). Both studies also highlighted the importance of patients feeling comfortable opening up to their therapists. The “Experiences of Therapy Over Time” theme here is like Hoskins et al.'s (2019) “Challenging but Beneficial” theme, where most patients found aspects of therapy difficult (challenging), but those who persisted reflected upon therapy as worthwhile (beneficial). Patterns in patients' experiences of CBT‐T and CBT‐AN‐20 may be explained by their similar therapeutic approaches, drawing on the inhibitory learning approach to exposure (Craske et al. 2014), which prioritizes maximizing safety learning in the long term over minimizing fear in the short term. This inherently anxiety‐inducing approach might, therefore, explain some of the difficult/challenging experiences patients report when addressing eating‐disordered behaviors and regaining weight. Cognitive‐behavioral theory (e.g., Greenberger and Padesky 2016; Williams and Garland 2002) suggests that as patients face these fears, their cognitions and emotions change, so they later perceive the early difficulties as worthwhile. This study highlights the importance of therapists supporting patients to face their fears.

### 4.2 Strengths and Limitations

The current study has strengths and limitations. Including both treatment completers and non‐completers provides a fuller understanding of patients' experiences of CBT‐AN‐20. Additionally, the analysts' lived experiences and independence from the intervention's design, delivery, and data collection processes bring a fresh perspective, helping future iterations of this novel therapy meet patients' needs (de la Rie et al. 2006; Hoskins et al. 2019). The methods of survey design and distribution were additional strengths. Designing the survey with input from people with lived experience ensured questions made sense from a patient perspective, and its distribution by someone other than their therapists facilitated open and honest feedback.

This study also has limitations. First, compared with interview‐based methods, using surveys may have limited the depth and richness of data collected in this study. While using survey data allows us to gain a snapshot of patients' experiences of CBT‐AN‐20 at this early stage of intervention development and testing, future research could build upon these preliminary findings by using interview methods to further explore patients' experiences. Second, this study's sample size (*N* = 16, 39.03% of 41 possible respondents) might limit the generalizability of its findings. While comparable with Hoskins et al.'s (2019) CBT‐T survey study (*N* = 17, 37.78% of 45 possible respondents), this study's sample was smaller than Toro et al. (2023; *N* = 24/47 possible respondents) and Zainal et al. (2016, *N* = 82/142 possible respondents). Third, respondents were self‐selected, potentially introducing bias and further limiting the generalizability of findings. While survey responses represented views from both treatment completers and non‐completers, it is unclear why some patients chose to respond to this survey, and some did not, and what perspectives are missing from this analysis. Fourth, given that participants came from a single study of CBT‐AN‐20 (Duggan et al. 2025, under consideration) is not possible to be sure that data saturation and generalizability to different settings were achieved. Therefore, other themes might arise in future research once CBT‐AN‐20 has been more widely used in clinical practice, and results are compared with those presented here. Fifth, there was a low IRR rate. This could be the result of genuine disagreement, from including multiple subthemes (increasing potential for disagreements), or a limitation of the IRR task. The inherent subjectivity in matching quotes with themes/subthemes may not lend itself to an accurate IRR calculation. However, this task facilitated discussions which resulted in 100% agreement on the final thematic analysis, supporting the reliability of results. Sixth, surveys were only issued to patients. Future research should also examine therapists' experiences to generate a more holistic picture, enabling comparisons between patients' and therapists' perspectives, and may seek perspectives of significant others. Seventh, participants were not invited to give feedback on this analysis, meaning that the analysts' interpretations might not match patients' intended meanings. Future research should include patients in checking the data analysis. Eighth, participants were predominantly young white females. Future research should include diverse patient groups where possible. Lastly, the analysts' lived experiences could be viewed as both a limitation and a strength. While potentially biasing the analysts' interpretation of data, their lived experience may also have facilitated a deeper understanding of patients' perspectives (Berger 2015; Dwyer and Buckle 2009). While co‐authors without lived experience reviewed the analysis, future research should consider including at least one analyst with no lived experience to include different perspectives.

### 4.3 Clinical Implications and Recommendations

First, results indicate that at least some adult outpatients with anorexia nervosa find CBT‐AN‐20 a feasible and acceptable therapy, supporting the promising empirical results in Duggan et al. (2025, under consideration). Therapists can therefore consider trialing CBT‐AN‐20 in clinical practice, as it works well for at least some patients. However, further research is needed to explore CBT‐AN‐20's effectiveness, including why some patients do well with this therapy, and some do not. This knowledge would enable more targeted individual treatment recommendations, and should identify potential aspects of the therapy for further development. Second, it would be helpful to provide clinicians with psychoeducational materials that they can share with patients in Session 1, to help set realistic expectations of CBT‐AN‐20 based on previous patients' experiences. Results suggested that the patients who benefited most from CBT‐AN‐20 were those who persisted through its difficult early stages to complete treatment, and who then reflected upon it as having been worthwhile. While we cannot draw any conclusions from this study as to why some patients completed treatment and others did not, survey responses suggest that patients differed in either their understanding or acceptance of this therapy's requirement for them to eat more and regain weight from the start of treatment (or at all). Psychoeducational materials could benefit from including quotes from previous patients who completed treatment, better explaining why eating more and regaining weight is worth doing, and showing that recovery is possible if patients persist and engage with the challenging early tasks of therapy. Starting treatment with open, honest conversations around how CBT‐AN‐20 can be “difficult but worthwhile” might help to improve patient retention and support the development of a positive therapeutic relationship.

## 5 Conclusions

CBT‐AN‐20 is a feasible and acceptable treatment for some adult outpatients with anorexia nervosa. Adding targeted psychoeducational materials could support patients through its challenging early stages. Future research should involve patients in the qualitative analysis and explore therapists' perspectives. Triangulating these results with feasibility, acceptability, and preliminary effectiveness data will direct the next steps in developing and testing this novel therapy, enhancing its potential to reduce waiting times and support more patients with anorexia nervosa to move toward recovery sooner.

## Author Contributions

**Heather C. Duggan:** methodology, data curation, formal analysis, validation, visualization, writing – original draft, writing – review and editing. **Charlotte Rose:** conceptualization, investigation, project administration, writing – review and editing, resources, supervision. **Hannah Turner:** resources, investigation, writing – review and editing, conceptualization, project administration, supervision. **Jessica Cox:** writing – review and editing, resources, investigation. **Rachel Ebbens:** resources, investigation, writing – review and editing. **Jade Southron:** validation, writing – review and editing. **Glenn Waller:** conceptualization, investigation, writing – review and editing, methodology, project administration, resources, supervision, visualization.

## Disclosure

We completed the Original Article checklist as described in the author guidelines.

## Ethics Statement

This study was cleared under UK National Health Service (NHS) Research and Development regulations, and ethical permission for data analysis was obtained from the University of Sheffield (064046).

## Conflicts of Interest

Three of the authors (C.R., H.T. and G.W.) will receive royalties from Routledge for the forthcoming manual. The other authors of this study have no conflicts of interest to declare.

## Supporting information


**Supporting Information A Table A.1**. Summary of Themes Identified in Previous Qualitative Studies of Patients' Experiences of Psychotherapies for Eating Disorders.

**Supporting Information B** CBT Evaluation Questionnaire.

**Supporting Information C** Instructions for Second Analyst.

**Supporting Information D** Quote Matching Activity, Interrater Reliability, and Kappa Calculations for “How Do Outpatients Experience 20‐Session Cognitive‐Behavioral Therapy for Anorexia Nervosa (CBT‐AN‐20)? A Qualitative exploration”.

**Supporting Information E Figure E.1**. Patients' Responses to Therapy Experience Questions on a 10‐Point Likert Scale (0 = Not at All, 10 = Completely/Extremely).

## Data Availability

The data that support the findings of this study are openly available in the University of Sheffield ORDA data repository at https://doi.org/10.15131/shef.data.27089422.
