# Supplementary material for: How Do Outpatients Experience 20‐Session Cognitive‐Behavioral Therapy for Anorexia Nervosa (CBT‐AN‐20)? A Qualitative Exploration
Source: Int J Eat Disord. 2025 Aug 21;58(11):2182–93. doi: 10.1002/eat.24528 (PMC12605776; doi:10.1002/eat.24528)
Supplement: Supplementary file 1 — Supporting Information A Table A.1. Summary of Themes Identified in Previous Qualitative Studies of Patients' Experiences of Psychotherapies for Eating Disorders. [file EAT-58-2182-s003.docx]

**Supporting Information A**

**Table A.1:** *Summary of Themes Identified in Previous Qualitative Studies of Patients’ Experiences of Psychotherapies for Eating Disorders.*

| **Author/s**  **Date** | ***N^a^*** | **Patient Diagnosis^b^** | **Intervention^c^** | **Method of Data Collection** | **Method of Data Analysis** | **Themes and Subthemes^d^** |
| --- | --- | --- | --- | --- | --- | --- |
| Isaksson et al.  2021 | 11 | Anorexia nervosa | Non-CBT  Radically open dialectical behavior therapy | Semi-structured interviews | Thematic analysis (Braun & Clarke, 2006) | A comprehensive treatment  *A flexible and complex approach*  *Following, or not following, the treatment*  *wholeheartedly*  *Skills for moving toward valued goals*  The benefits of sharing and connecting with others  *A trusting and genuine therapeutic*  *relationship*  *Sharing and connecting with others in the*  *group*  Growing trust  *Initial skepticism*  *Change takes time*  Moving towards valued goals -but some remain  *Getting to know myself in a kind, but*  *sometimes painful, way*  *A journey from rigidity to more flexibility,*  *openness, and connectedness*  *Changes in the eating disorder*  *... But some remain*  Doing well in treatment  *Not wanting to be a bother*  *Being the best of patients* |
| Lose et al.  2014* | 17 | Anorexia nervosa | Non-CBT  Maudsley Model for Treatment of Adults with Anorexia Nervosa (MANTRA) and Specialist Supportive Clinical Management (SSCM) | Semi-structured interviews | Thematic analysis (Braun & Clarke, 2006) | MANTRA:  Positive and helpful aspects  *Structured and flexible approach*  *Helpful manual*  *Other helpful aspects*  Beneficial outcomes  *Altered stance to feelings and thought*  *processes*  *Improved communication and confidence*  *Improved quality of life*  Less helpful aspects  *Issues with the manual and treatment*  Possible improvements  *Changes to the treatment, manual and*  *frequency of sessions*  *Practical advice and prescriptiveness*  SSCM:  Positive and helpful aspects  *Practical and proactive approach*  *Focus on nutrition*  Beneficial outcomes  *Learning to move forward*  *Understanding ED symptoms and their*  *impact on behavior*  *Improved communication and confidence*  *Improved quality of life*  Less helpful aspects  *Focus on weight and eating*  *Limited therapeutic content, continuity and*  *structure*  Possible improvements  *Changes to therapeutic content, process and*  *structure*  *Treatment duration and intensity*  Both therapies:  Positive and helpful aspects  *Regularity and predictability*  *Talking therapy*  Less helpful aspects  *Delays and disruptions*  Therapeutic and external environment  *Therapist match to patient*  *Therapist’s characteristics and approach*  *External circumstances* |
| Rennick et al.  2024 | 16** | Anorexia nervosa | Non-CBT  Specialist Psychotherapy with Emotion for Anorexia in Kent and Sussex (SPEAKS) | Semi-structured interviews | Reflexive thematic analysis (Braun & Clarke, 2006; 2019; 2021) | Shift in treatment focus and experience  *Appreciation for creating space from their*  *eating disorder*  *Focus on emotions*  *Uncertainty around experiential techniques*  Balancing resources and treatment outcomes  *Worries over amount of resource needed to*  *deliver SPEAKS*  *Frequency and duration allowing for long*  *term change*  *Confidence with moving forwards*  *independently*  Navigating the online treatment environment  *Not being able to immerse in the therapy*  *experience*  *The ease of online sessions*  *Valuing the opportunity to connect with*  *colleagues*  Therapist adaptation and professional development  *Confidence with new techniques takes time*  *Appreciation for regular supervision and*  *training*  Research processes  *Future randomized control trial (RCT)*  *Research components*  *Emotional responses to involvement in a*  *new intervention* |
| Zainal et al. 2016* | 82 | Anorexia nervosa | Non-CBT  Maudsley Model for Treatment of Adults with Anorexia Nervosa (MANTRA) and Specialist Supportive Clinical Management (SSCM) | Written feedback forms | Thematic analysis (Braun & Clarke, 2006) | MANTRA:  Treatment aspects  *Use of manual*  *Treatment focus*  Treatment outcomes and recovery  *Positive outcomes*  *Negative outcomes*  *Stage of recovery*  SSCM:  Treatment aspects  *Lack of structure*  *Treatment focus*  Treatment outcomes and recovery  *Positive outcomes*  *Negative outcomes*  *Stage of recovery*  Both therapies:  *Duration, frequency and disruptions*  *External social support*  *Pacing and individualization of treatment*  *Therapist*  *Concerns about ending therapy* |
| Hoskins et al.  2019 | 17 | Other non-underweight eating disorder | CBT  CBT-T | Survey (questionnaire) | Thematic analysis (Braun & Clarke, 2006) | Therapeutic relationship  *Therapist characteristics*  *Felt comfortable in therapy*  *No longer alone,*  *Firm but fair*  Nature of therapy  *Timing*  *Personalization*  *Personal effort*  *Therapy structure and intervention*  Challenging but beneficial  *Hard but necessary*  *Challenges*  *Initial skepticism*  Ending therapy  *Outcome*  *After therapy*  Overall experience of therapy  *Comparison to other therapies*  *Compliments* |
| McClay et al.  2013 | 8 | Other non-underweight eating disorder | CBT  Overcoming Bulimia Online | Semi-structured interviews | Thematic analysis (Braun & Clarke, 2006) | Conceptualizing eating disorders  *Impact of and feelings about bulimia*  *Perceptions of eating disorders/people with*  *eating disorders*  *Acknowledgement of/acceptance of the*  *problem*  Help-seeking behavior  *Past experiences*  *Barriers to help*  *Reasons for choosing self-help*  *Prior knowledge*  The intervention  *Support worker*  *Positive aspects*  *Negative aspects/difficulties*  Motivation  *Aids*  *Challenges*  Participant engagement  *Opportunity to help self and others*  *Experience as an online research*  *participant*  Progress and recovery  *Improvements in bulimic symptoms*  Privacy  *Secrecy*  *Talking about bulimia* |
| Sánchez-Ortiz et al.  2011 | 9 | Other non-underweight eating disorder | CBT  iCBT - Overcoming Bulimia Online | Semi-structured interviews | Thematic analysis (Braun & Clarke, 2006) | Reasons for choosing this form of treatment  Experiences of treatment  *Confidentiality/privacy*  *Flexibility*  *Ease of use*  *Feeling supported – including help with*  *motivation*  *Content of program*  Impact of treatment  *Expectations about outcome*  *Effectiveness – changes in ED symptoms*  *Effectiveness – other changes*  *Tools for coping in the future*  Comparison between iCBT and other forms of treatment  *Counseling*  *General practitioner (GP)*  *Other forms of self-help*  Feedback  *Timing*  *Other methods of support* |
| Toro et al.  2023 | 24 | Other non-underweight eating disorder | CBT  CBT-T (in workplace setting) | Survey (questionnaire) | Thematic analysis (Braun & Clarke, 2006) | Work or the workplace as a trigger  *Demanding workload or work-related*  *stress*  *Food availability at work*  *Social pressure and comparison*  *Poor relationships at work*  Pandemic and related changes to work environment  *General impact of pandemic and working*  *from home*  *Availability of food*  *Lack of breaks*  *Loneliness*  *Changes to exercise regime*  Accessibility of workplace therapy  *Convenient (saves time/money)*  *Privacy for sessions*  *Less intimidating than other therapy*  *settings*  *Fear of stigma*  *Personal preference*  Work environment facilitated engagement  *Working from home*  *Supportive manager and/or colleagues*  *Access to food at work*  *Routine of the workplace*  *Workplace advertisements motivating to*  *seek support*  Impact of therapy on work  *Focus and productivity*  *No benefits seen*  *Less preoccupation with eating disorder*  Impacts of therapy on the self  *Reduced preoccupation with food and/or*  *body image*  *Increased energy levels*  *Mood improvements*  *Sustainability of change during follow-up*  *Achievement of personal goals*  *Improved control over food*  Role of the therapist  *Therapeutic relationship and firm empathy*  *approach*  *Tailored approach* |
| Traviss et al.  2011 | 7*** | Other non-underweight eating disorder | CBT | Semi-structured interviews | Thematic framework analysis (Richie & Spencer, 1994) | Necessity of having a guide as a facilitator  *Roles*  *Facilitator not therapist*  *Monitoring*  *Motivating*  *Supporting*  *Challenging behaviors*  *Skills*  *Using additional skills to manage*  *difficulties*  *Tailoring materials*  *Assessing suitability*  Therapeutic relationship - clients with positive outcomes  *Characteristics of the relationship*  *Strong and developed*  *Open*  *Collaborative*  *Guide quality*  *Respectful and non-judgmental*  *Interested*  *Personal and emotional detachment*  *Flexible and responsive*  *Guide skills in managing the relationship*  *Able to identify ruptures*  *Client characteristics*  *Realistic expectations*  Therapeutic relationship - clients with poor outcomes  *Characteristics of the relationship*  *Deteriorated*  *Good but client not suitable*  *Good but client cured*  *Negative client characteristics*  *Reticent*  *Resistant*  *False expectations*  *Psychological disturbance*  *Rupture*  *Betrayal by guide*  *Interruption to relationship*  Client suitability  *Readiness*  *Eating disorder diagnosis*  *Severity of eating disorder*  *Level of psychological disturbance*  *Problems outside of therapy*  *Having sufficient time*  *Willingness to take responsibility*  *Treatment preference* |

*Note.* *While separate studies, both Lose et al. (2014) and Zainal et al. (2016) were conducted as part of the same larger trial. *This study included six therapists in addition to the 16 patients, so the themes reported are reflective of both groups of participants.*** This study included five guides in addition to the seven clients, so the themes reported are reflective of both groups of participants.

^a^*N* = The number of patients who provided data for qualitative analysis (not necessarily the total number of participants).

^b^Anorexia nervosa/Other non-underweight eating disorder (e.g., bulimia nervosa, binge eating disorder, eating disorders not otherwise specified).

^c^CBT/Non-CBT, with the specific names of interventions stated.

^d^Subthemes and sub-subthemes are italicized and indented.

**References**

Braun, V., & Clarke, V. (2006). Using thematic analysis in psychology. *Qualitative Research in Psychology*, *3*(2), 77–101. https://doi.org/10.1191/1478088706qp063oa

Braun, V., & Clarke, V. (2019). Reflecting on reflexive thematic analysis. *Qualitative Research in Sport, Exercise and Health, 11*(4), 589–597. https://doi.org/10.1080/2159676X.2019.1628806

Braun, V., & Clarke, V. (2021). One size fits all? What counts as quality practice in (reflexive) thematic analysis? *Qualitative Research in Psychology, 18*(3), 328–352. https://doi.org/10.1080/14780887.2020.1769238

Hoskins, J. I., Blood, L., Stokes, H. R., Tatham, M., Waller, G., & Turner, H. (2019). Patients' experiences of brief cognitive behavioral therapy for eating disorders: A qualitative investigation. *International Journal of Eating Disorders*,  *52*(5), 530–537. https://doi.org/10.1002/eat.23039

Isaksson, M., Ghaderi, A., Wolf-Arehult, M., Öster, C., & Ramklint, M. (2021). Sharing and connecting with others – patient experiences of radically open dialectical behavior therapy for anorexia nervosa and overcontrol: A qualitative study. *Journal of Eating Disorders*, *9*(1), 29. https://doi.org/10.1186/s40337-021-00382-z

Lose, A., Davies, C., Renwick, B., Kenyon, M., Treasure, J., & Schmidt, U. (2014). Process evaluation of the Maudsley Model for Treatment of Adults with Anorexia Nervosa trial. Part II: Patient experiences of two psychological therapies for treatment of anorexia nervosa. *European Eating Disorders Review*, *22*(2), 131–139. https://doi.org/10.1002/erv.2279

McClay, C. A., Waters, L., McHale, C., Schmidt, U., & Williams, C. (2013). Online cognitive behavioral therapy for bulimic type disorders, delivered in the community by a nonclinician: Qualitative study. *Journal of Medical Internet Research*, *15*(3), e46–e221. https://doi.org/10.2196/jmir.2083

Rennick, A., Papastavrou Brooks, C., Singh Basra, R., Startup, H., Lavender, T., & Oldershaw, A. (2024). Acceptability of Specialist Psychotherapy with Emotion for Anorexia in Kent and Sussex (SPEAKS): A novel intervention for anorexia nervosa. *International Journal of Eating Disorders*, *57*(3), 611–623. https://doi.org/10.1002/eat.24139

Ritchie, J., & Spencer, L. (1994). Qualitative data analysis for applied policy research. In A. Bryman & R. G. Burgess (Eds.), *Analysing qualitative data* (pp. 173–194). Routledge.

Sánchez-Ortiz, V. C., House, J., Munro. C., Treasure, J., Startup, H., Williams, C. & Schmidt., U. (2011). “A computer isn’t gonna judge you”: A qualitative study of users’ views of an internet-based cognitive behavioural guided self-care treatment package for bulimia nervosa and related disorders. *Eating and Weight Disorders - Studies on Anorexia, Bulimia and Obesity, 16*(2), e93-e101. https://doi.org/10.1007/BF03325314

Toro, C. T., Payne, A., Jackson, T., Russell, S., Daly, G., Waller, G., & Meyer, C. (2023). Evidence for feasibility of implementing online brief cognitive‐behavioral therapy for eating disorder pathology in the workplace. *International Journal for Eating Disorders, 56*(6), 1254-1268. https://doi.org/10.1002/eat.23961

Traviss, G. D., Heywood-Everett, S., & Hill, A. J. (2011). Understanding the ‘guide’ in guided self-help for disordered eating: A qualitative process study. *Psychology and Psychotherapy: Theory, Research and Practice, 86*(1), 86–104. doi.org/10.1111/j.2044-8341.2011.02049.x

Zainal, K. A., Renwick, B., Keyes, A., Lose, A., Kenyon, M., DeJong, H., Broadbent, H., Serpell, L., Richards, L., Johnson-Sabine, E., Boughton, N., Whitehead, L., Treasure, J., & Schmidt, U. (2016). Process evaluation of the MOSAIC trial: Treatment experience of two psychological therapies for out-patient treatment of anorexia nervosa. *Journal of Eating Disorders*, *4*(1), 2. https://doi.org/10.1186/s40337-016-0091-5
