# Supplementary material for: How Do Outpatients Experience 20‐Session Cognitive‐Behavioral Therapy for Anorexia Nervosa (CBT‐AN‐20)? A Qualitative Exploration
Source: Int J Eat Disord. 2025 Aug 21;58(11):2182–93. doi: 10.1002/eat.24528 (PMC12605776; doi:10.1002/eat.24528)
Supplement: Supplementary file 2 — Supporting Information B CBT Evaluation Questionnaire. [file EAT-58-2182-s005.docx]

**Supporting Information B**

**CBT Evaluation Questionnaire**

Did you have *previous* therapy for an eating disorder?

□Yes □No

If yes, how many courses of therapy did you have? _______

**In relation to your most recent experience of CBT**

**How many sessions did you attend? ______**

**How suitable did the treatment feel for you as an individual?** (please circle)

0--------1--------2--------3--------4--------5---------6---------7---------8--------9--------10

not at all completely suitable suitable

**How much do you feel the treatment helped you to reduce your eating disorder behaviours?** (please circle)

0--------1--------2--------3--------4--------5---------6---------7---------8---------9---------10

not at all completely

**How much do you feel the treatment helped you to reduce your eating disorder thinking patterns?** (please circle)

0--------1--------2--------3--------4--------5---------6---------7---------8---------9---------10

not at all completely

**How much do you feel the treatment helped you to improve your body image?** (please circle)

0--------1--------2--------3--------4--------5---------6---------7---------8---------9---------10

not at all completely

**How much do you feel the treatment helped you to improve your emotional state?** (please circle)

0--------1--------2--------3--------4--------5---------6---------7---------8---------9---------10

not at all completely

**How much do you feel the treatment helped you move towards recovery?** (please circle)

0--------1--------2--------3--------4--------5---------6---------7---------8---------9---------10

not at all completely

**How much do you feel the treatment helped you to improve your quality of life?** (please circle)

0--------1--------2--------3--------4--------5---------6---------7---------8---------9---------10

not at all completely

**Please describe your overall experience of this course of therapy**

-----------------------------------------------------------------------------------------------------------------------------------------------------------------------------------------------------------------------------------------------------------------------------------------------------------------------------------------------------------------------------------------------------------------------------------------------------------------------------------------------------------------------------------------------------------------------------------------------------------------------------------------------------------------------------------------------

**What did you find most helpful about treatment and why?**

----------------------------------------------------------------------------------------------------------------------------------------------------------------------------------------------------------------------------------------------------------------------------------------------------------------------------------------------------------------------------------------------------------------------------------------------------------------------------------------------------------------------------------------------------------------------------------------------------------------------------------------------------------------------------------------------------------------------------------------------------------------------------------------------------------------------------------------------------------------------------------------------------

**What did you find least helpful about treatment and why?**

--------------------------------------------------------------------------------------------------------------------------------------------------------------------------------------------------------------------------------------------------------------------------------------------------------------------------------------------------------------------------------------------------------------------------------------------------------------------------------------------------------------------------------------------------------------------------------------------------------------------------------------------------------------------------------------------------------------------------------------------------------------------------------------------------------------------------------------------------------------------------------------------------

**Were there any parts of therapy that you found more difficult – what were they, and why were they difficult?**

------------------------------------------------------------------------------------------------------------------------------------------------------------------------------------------------------------------------------------------------------------------------------------------------------------------------------------------------------------------------------------------------------------------------------------------------------------------------------------------------------------------------------------------------------------------------------------------------------------------------------------------------------------------------------------------------------------------------------------------------------------------------

**Please tell us about the relationship that you had with your therapist.**

------------------------------------------------------------------------------------------------------------------------------------------------------------------------------------------------------------------------------------------------------------------------------------------------------------------------------------------------------------------------------------------------------------------------------------------------------------------------------------------------------------------------------------------------------------------------------------------------------------------------------------------------------------------------------------------------------------------------------------------------------------------------

**If you’ve had any previous therapy how did your experience of this course compare to previous courses?**

------------------------------------------------------------------------------------------------------------------------------------------------------------------------------------------------------------------------------------------------------------------------------------------------------------------------------------------------------------------------------------------------------------------------------------------------------------------------------------------------------------------------------------------------------------------------------------------------------------------------------------------------------------------------------------------------------------------------------------------------------------------------

**Do you have any comments or suggestions about how this specific therapy might be improved?**

------------------------------------------------------------------------------------------------------------------------------------------------------------------------------------------------------------------------------------------------------------------------------------------------------------------------------------------------------------------------------------------------------------------------------------------------------------------------------------------------------------------------------------------------------------------------------------------------------------------------------------------------------------------------------------------------------------------------------------------------------------------------

**How likely is it that you would recommend this therapy to someone with similar eating difficulties to you?**

0--------1--------2--------3--------4--------5---------6---------7---------8---------9---------10

not at all Extremely

likely likely

| **About you**  We request the following demographic details to inform our understanding of how accessible and suitable the intervention has been for a diverse range of people. We also recognise and respect that some people prefer not to share certain information about themselves, if this applies to you then please leave any relevant sections leave blank. | |
| --- | --- |
| **Age**: __________________  **Gender:** _______________  **Sex (assigned at birth):**  □Female  □Male  □Intersex | **Ethnicity:** ____________­­­­________  **Employment status:**  □Employed  □Unemployed □Student  □Homemaker □Retired  □Other (please specify)______________ |

**Thank you for your time and insights into your experience of therapy. This will help us to shape therapies for others in the future.**
