# Supplementary material for: How Do Outpatients Experience 20‐Session Cognitive‐Behavioral Therapy for Anorexia Nervosa (CBT‐AN‐20)? A Qualitative Exploration
Source: Int J Eat Disord. 2025 Aug 21;58(11):2182–93. doi: 10.1002/eat.24528 (PMC12605776; doi:10.1002/eat.24528)
Supplement: Supplementary file 3 — Supporting Information C Instructions for Second Analyst. [file EAT-58-2182-s002.docx]

**Supporting Information C**

**Instructions for Second Analyst**

First, thank you again for participating in this research project. We aim to analyse 16 patients’ responses to a survey after receiving a brief cognitive-behavioural therapy for anorexia nervosa, named CBT-AN-20, as part of a pilot study of this novel therapy. To analyse patients’ qualitative (written) responses, we are using thematic analysis, which seeks to identify common themes or patterns in patients’ responses that address our two research questions:

*1. What can we learn about patients’ experiences of CBT-AN-20?*

*2. What do patients’ experiences tell us about how we might do therapy better in future?*

Heather Duggan, the main analyst, has followed a systematic process for identifying themes, subthemes (i.e., more specific themes within the context of a broader theme), and sub-subthemes (i.e., even more specific themes within the context of a subtheme). The results are presented in a thematic map (Figure 1, page 3). Its branches show that sub-subthemes are nested within subthemes, which are nested within three broad themes. Namely, “Importance of Therapeutic Relationship”, “Experiences of Therapy Over Time”, and “Nature of Therapy”.

At this point, the thematic map is not final. Your input is invaluable in ensuring that the final thematic map accurately represents patients’ survey responses and that the proposed themes, subthemes, and sub-subthemes make sense from the perspective of a person with lived experience of an eating disorder.

Your tasks are to:

1. Read the survey questions and transcript of patients’ responses to familiarise yourself with the data we are analysing. Make a note of any initial observations you have, including what themes/patterns you spot in patients’ responses.
2. Review the thematic map to familiarise yourself with the main analyst’s proposed themes, subthemes, and sub-subthemes (Figure 1, page 3).
3. Complete the quote-matching exercise in the Excel spreadsheet by matching patients’ quotes with the theme, subtheme, and/or sub-subtheme that you think best fits. This exercise has been designed to help us evaluate whether the proposed thematic map makes sense and fits with the data.
4. Return the completed spreadsheet by the date agreed in your first meeting to Heather Duggan at [hduggan1@sheffield.ac.uk](mailto:hduggan1@sheffield.ac.uk).
5. After completing the matching task, please also reflect on how well you think the thematic map represents the data. Questions to help you reflect:

- What works/does not work about the map, and why?
- Is there anything about the map that needs to be added or clarified? If so, what is it, and how do you think we can improve the map?
- Do the theme names work? Why, or why not? Note any suggestions you have for better names.
- Note any further thoughts, ideas, or points to discuss in the next meeting.

1. Bring any notes on your initial observations and reflections after completing the activity to our next meeting for discussion.

We will arrange a suitable time and date for our second meeting when we first meet to allow you enough time to complete the above tasks and return the spreadsheet for review. As a rough estimate, we suggest that these activities may take you about 2-3 hours to complete, but the exact amount of time you spend is up to you. The second meeting will be about 1-1 ½ hours long. We will look at where we agree and disagree on the quote-matching tasks, discuss any notes you have made, evaluate the proposed thematic map, and agree upon any adjustments that need to be made before it is published in a paper.

We would also like to thank you again for your help and participation in this by including your name as an author on the final published paper if you are happy to be included. This is, of course, up to you, and it is absolutely fine if you prefer to remain anonymous

**Figure 1:** Thematic Map

Does One Size Fit All?

Comparisons with Previous Therapies

Limited Duration of Therapy

Nature of Therapy

Importance of Therapeutic Relationship

Perceptions of Communication Shape Connection

Therapist Qualities Matter

Good/Bad Relationships

Exposure

Difficult but Worthwhile

**Patient Experiences of CBT-AN-20:**

What can we learn and what might we do better in future?

Therapy was Acceptable

Therapy Worked

Weight and Weighing

Food and Eating

Cognitive Restructuring

Problem-Solving

Hopeful about Recovery

Therapy was Worthwhile

Tracking Food and Eating

Weight and Weighing

Food and Eating

Weight and Weighing

Therapy was Unhelpful

Experiences of Therapy Over Time

Therapy was Difficult
