# Supplementary material for: How Do Outpatients Experience 20‐Session Cognitive‐Behavioral Therapy for Anorexia Nervosa (CBT‐AN‐20)? A Qualitative Exploration
Source: Int J Eat Disord. 2025 Aug 21;58(11):2182–93. doi: 10.1002/eat.24528 (PMC12605776; doi:10.1002/eat.24528)
Supplement: Supplementary file 5 — Supporting Information E Figure E.1. Patients' Responses to Therapy Experience Questions on a 10‐Point Likert Scale (0 = Not at All, 10 = Completely/Extremely). [file EAT-58-2182-s001.docx]

**Supporting Information E**

**Figure E.1**

*Patients’ Responses to Therapy Experience Questions on a 10-Point Likert Scale (0 = Not at All, 10 = Completely/Extremely).*


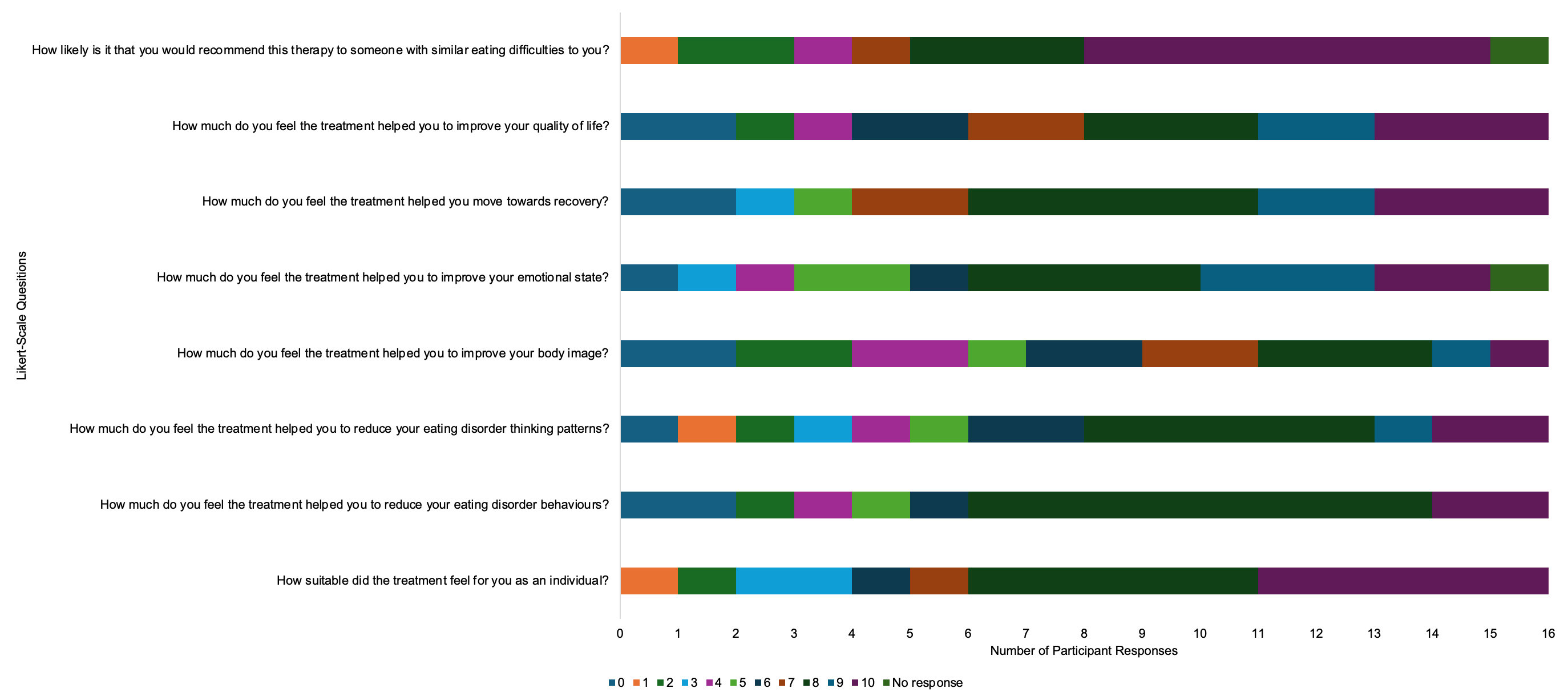
*Note. N* = 16.
